# Assessment of Control Measures and Trends of Malaria in Burie-Zuria District, West Gojjam Zone, Amhara Region, North West Ethiopia

**DOI:** 10.1155/2015/302194

**Published:** 2015-06-11

**Authors:** Addisu Workineh Kassa, Mulugojjam Andualem Tamiru, Addisu Gize Yeshanew

**Affiliations:** ^1^Amhara Regional Health Bureau, Bahir Dar, Ethiopia; ^2^Bahir Dar University, Bahir Dar, Ethiopia; ^3^Jimma University, Jimma, Ethiopia

## Abstract

*Introduction*. Malaria is caused by the protozoan parasite *Plasmodium* and transmitted by the bite of *Anopheles* mosquitoes. The aim of this study was to assess control measures and trends of malaria and guide intervention measures at Burie-Zuria district, Amhara region. *Methods*. Descriptive cross-sectional assessment of control measures was undertaken. We used health facility records of malaria data. We surveyed households for clinical malaria cases and utilization of Long Lasting Impregnated Nets (LLINs) and its status; the condition of Indore Residual Spraying (IRS) operation at household level was observed. *Results*. In Zelma-Shenbekuma kebele (village) the prevalence rate of confirmed malaria cases in the 2nd week of September was 1.2 per 1000 (17) of population and increased to 11.5 per 1000 (163) of population in the 3rd week of September 2012 and reached 16.6 per 1000 (236) of population in the 1st week of November 2012. The attack rate was the highest in 1-<5 years 120.3 per 1000 (1920) of population. LLINs were distributed four years back and only five of the fifteen respondents knew about the use of LLINs and used it regularly. Four of the fifteen households were not sprayed with IRS. *Conclusion*. Vector control interventions were not carried out timely.

## 1. Introduction

Malaria remains to be one of the main global health problems of our time, causing more than one million deaths per year, with about 90% of deaths and 60% of cases occurring in Africa south of the Sahara. It is caused by the protozoan parasite* Plasmodium* and transmitted by* Anopheles* mosquitoes, which bite mainly between sunset and sunrise [1].

Approximately 54 (68%) million people live in malaria risk areas in Ethiopia, primarily at altitudes below 2,000 meters. Malaria is mainly seasonal with unstable transmission in the highland fringe areas and of relatively longer transmission duration in lowland areas, river basins, and valleys.* Anopheles arabiensis* is the main malaria vector;* Anopheles pharoensis, Anopheles funestus,* and* Anopheles nili* play a role as secondary vectors [2–4].

Improved epidemic preparedness greatly reduces the risk of recurrent epidemics [3, 5]. Indoor residual spraying (IRS) in Ethiopia has been one of the most popular interventions for malaria control. Indoor residual spraying (IRS) requires high coverage if any protection is to be achieved, and the required coverage must be achieved before and throughout the transmission season. Timeliness is particularly important in the control of outbreaks. Effective coverage should be maintained during the entire transmission season which implies that spraying of the whole area to be protected should be completed before the beginning of that season [6–8].

**Figure 1:**
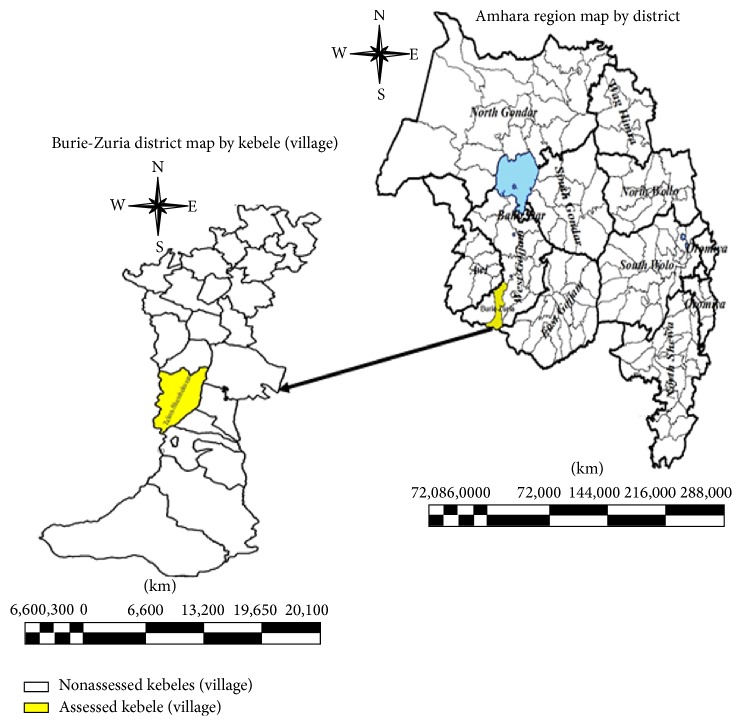
Burie-Zuria district map with its kebeles (villages).

Malaria remains to be the major public health challenge in Amhara region. Among ten zones and three town administrations in Amhara region, West Gojjam zone accounts for one-third of the malaria burden of the region [3, 9]. This zone constitutes 15 districts, among which half of the malaria burden exists just in three districts: Womberma (24%), Burie-Zuria (13%), and Jabi Tehnan (12%). Burie-Zuria district is one of the largest districts in West Gojjam zone with population of 118,020. It constitutes 20 kebeles (villages) with 24 health facilities (19 health posts and five health centers) (Figure 1). All kebeles (villages) in the district are considered to be malarious.

Investigation was conducted in collaboration with district health office and zonal heath department to confirm and verify outbreaks of malaria and implement control and preventive measures.

The aim of investigation was to investigate the occurrence of malaria outbreak, identify the risk factors, and suggest practical control measures to alleviate the disease burden of the community in Zelma-Shenbekuma kebele (village) of Burie-Zuria district.

## 2. Methodology

### 2.1. Study Area

Burie-Zuria district is one of the malaria endemic districts which covers 587.95 sq.km and has 19 rural and one urban kebeles. Rainfall ranges from 1500 to 2000 mm per annum and 13.3% of the district is Kola, 66% is Woina Dega, and 20.7% of the area is Dega. The total population estimate of the district was 101,319 and Zelma-Shenbekuma kebele (village) has a population of 14,188 for 2012/13 [10].

### 2.2. Study Design

Descriptive cross-sectional study was conducted in the Zelma-Shenbekuma kebele (village) of Burie-Zuria district.

#### 2.2.1. Standard Case Definition of Malaria

The standard case definition was any person with fever or history of fever and confirmed with microscopy or rapid diagnostic tests (RDTs) for malaria parasites [5].

### 2.3. Data Collection Method

We used semistructured questionnaire to collect the necessary information. We used health facility records of malaria data from daily registers. We discussed with health extension workers (HEWs) and we collected the available morbidity, mortality, and diagnostic data. We surveyed households for clinical malaria cases and utilization of Long Lasting Impregnated Nets (LLINs) and its status; the condition of indoor residual spraying (IRS) was interviewed households using semistructured questionnaire in order to collect risk factors for the malaria outbreak.

We compiled daily data to weekly total cases in the affected locality by taking the 3rd quartile or the 2nd largest number from the past five years (2007–2011) on weekly basis and compared with the number of cases of the year 2012. The available daily data was compared with the same week of the threshold to define the rapid increase of malaria cases.

#### 2.3.1. Observation

Utilization of Long Lasting Impregnated Nets (LLINs) and its status and the condition of indoor residual spraying (IRS) operation at household level was observed. Mosquito breeding sites were also visited in the kebele (village).

### 2.4. Data Collection Period

Data was collected from 25/10/2012 to 28/10/2012.

### 2.5. Data Analysis

The collected data was checked for completeness for each variable and subsequently entered into a computer after transcribing from a hard copy which is used to collect the raw data from registries. Then the data has been cleaned and analyzed using Microsoft Excel. Prominent findings of the analysis were presented in figures.

### 2.6. Ethical Review

The study was conducted in accordance with the national policies on ethics. Ethical approval was obtained from the Research Ethical Review Committee of Amhara Regional Health Bureau and from selected district health office and oral consent was asked for from health extension workers.

## 3. Results

Estimated total population of 14,188 was at risk of malaria in Zelma-Shenbekuma kebele (village).

There were three health extension workers (HEWs) in Zelma-Shenbekuma kebele (village) health post. There were a total of 1,039 confirmed malaria cases out of 1549 rapid diagnostic tests (RDTs) done in eight weeks from daily records from September to 28/10/2012, out of which 48.9% (508) were females; in each week between 17 and 236 confirmed cases were treated.

**Figure 2:**
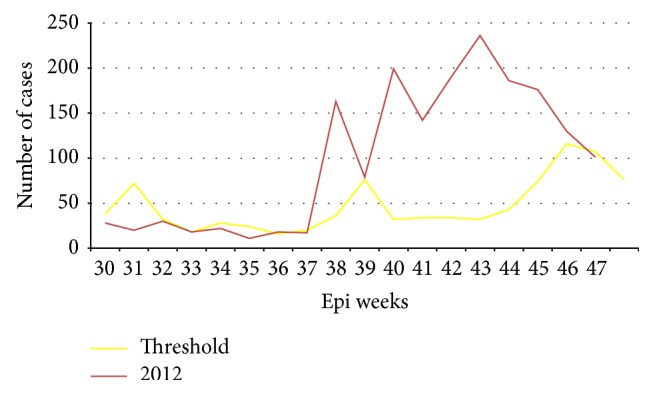
Confirmed malaria cases of Zelma-Shenbekuma kebele (village) from July to November 2012.

**Figure 3:**
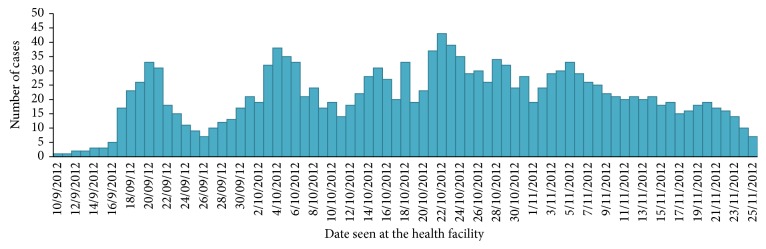
Epi curve of malaria outbreak by date of visit in Zelma-Shenbekuma (village) health post, Burie-Zuria, Amhara, Ethiopia, from September to November 2012.

**Figure 4:**
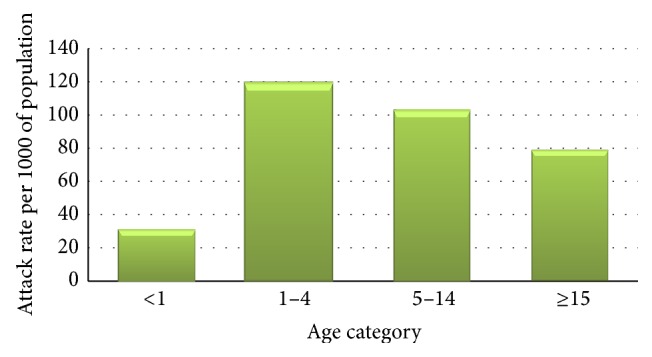
Confirmed malaria cases in age category of Zelma-Shenbekuma kebele (village) from September to November 2012.

The prevalence rate of confirmed malaria cases in the 2nd week of September was 1.2 per 1000 (17) of population and increased to 11.5 per 1000 (163) of population in the 3rd week of September 2012 and reached 16.6 per 1000 (236) of population in the 1st week of November 2012. The attack rate was the highest in 1–<5 years 120.3 per 1000 (1920) of population followed by 5 to < 15 years 103.4 (3761), 79.1 (8154) for ≥15 years, and 31.2 (353) per 1000 of population for under one year with no sex difference (Figures 2, 3, and 4).

### 3.1. Vector Control Interventions

Indoor residual spraying (IRS) with deltamethrin spray with expected 100% coverage of all households was carried out in the affected kebele (village) in October 2012. Long Lasting Impregnated Nets (LLINs) were distributed four years back and only some of the LLINs were replaced in July 2012 due to the shortage of LLINs for replacement.

### 3.2. Observation

Among the fifteen households visited randomly, in eleven households, there were one to three household members diagnosed with malaria at the health post. The team noted that four of the fifteen households were not sprayed for various reasons though the household owners were at home during spray operation. This suggested that indoor residual spraying (IRS) quality and efficiency was found doubtful as spray men were expected to spray all households in the kebele as long as the household owners were there. Only five of the fifteen respondents reported that they know about the use of Long Lasting Impregnated Nets (LLINs) and that they are using it regularly. Others perceived that it does not prevent malaria. Eleven of the visited households had LLINs hanged over the beds. However, nets in seven of fifteen visited households had tears/holes.

### 3.3. Interventions Taken

The following control and prevention measures were carried out during the field visit:

- Case management was carried out with coartem and chloroquine.
- We used public conferences to address the issue and promote Long Lasting Impregnated Nets (LLINs) utilization and environmental management activities.
- We teach health extension workers (HEWs) and district health office supervisors to advocate the use and mending of Long Lasting Impregnated Nets (LLINs) until replacement.
- Switching the use of deltamethrin to propoxur for indoor residual spraying (IRS) in remaining kebeles (villages) was carried out.
- Partners were requested to assist district health office in intensifying active case surveillance.

## 4. Discussion

To assess control measures and trends of malaria in Zelma-Shenbekuma kebele (village), we used five years' data (2007–2011) of the 3rd quartile or the 2nd largest number as a threshold to compare with 2012 on weekly basis. We identified the outbreaks of malaria and ongoing outbreak during assessment in the kebele (village). The number of malaria cases crossed the threshold starting from the 2nd week of September. The health post had weekly malaria epidemic monitoring chart for early detection of epidemics but they were not reported to the district health office when the number of cases crossed the threshold.

It was an exaggerated seasonal increase precipitated by interruption of vector control activities. There were multiple breeding sites difficult for environmental management (Fetem-West and Quamit-East rivers) which surround the kebele and might be the source of the outbreak. Moreover indoor residual spraying of houses in affected kebele with deltamethrin was not performed timely, sprayed after the outbreak begun.

Study in Burundi showed that sleeping under a net reduced the prevalence of malaria by 64% while indoor residual spraying (IRS) had no effect [4]. Malaria transmission in Ethiopia's seasonal epidemiological settings usually ends in December; indoor residual spraying (IRS) coverage must be achieved before and maintained during the transmission season. Malaria cases were not declined rapidly even after the area was sprayed; this might be the resistance of* Anopheles *mosquitoes to deltamethrin. Other vector control measures such as Long Lasting Impregnated Nets (LLINs) were not replaced timely and Shenbekuma got (subkebele) not replaced at all in which most of the cases came from, among nine gots of the kebele. Abet chemical not sprayed for removing larvae might result in seasonal peaks to levels that surpass the epidemic threshold. There was no intervention taken by community in the year 2012 and health extension workers (HEWs) did not work on environmental management (environmental manipulation or modification). The team explained the wide spreading outbreak situation in the kebele and what the district administrator needs to do in mobilizing the community and addressing prevention activities through kebele leaders and Health Development Army (HDA). Administrators' role is vital in health education and improving health seeking behavior; the team explained what needs to be done in view of the required political leadership in terms of supporting the health extension workers (HEWs).

## 5. Conclusions and Recommendations

The health post managing malaria cases uses malaria diagnostic service based on national protocol. The attack rate was the highest among 1–<5 years (120.3 per 1000 of population) compared to the other age groups with no sex difference. Vector control interventions were not carried out timely in the kebele. The collected data from the health facility showed outbreaks before they announced to the district health office that the number of cases crossed the threshold. The district started response to the outbreak lagged by one week after announcing and assigning additional health professionals from the cluster health center for case management. To prevent subsequent malaria outbreaks in the kebele (village) the following action points were put as recommendation:

- Timely indoor residual spraying (IRS) operation should be done with appropriate chemical before transmission seasons.
- Worn-out Long Lasting Impregnated Nets (LLINs) should be replaced timely.
- Health extension workers and district health offices should advocate the use and mending of Long Lasting Impregnated Nets (LLINs) until replacement is done.
- Keep weekly malaria data and use it for early detection of malaria outbreaks.
- Monitoring and follow-up of health facilities has to be strengthened.

## References

[1] World Health Organization (2004). *Malaria Elimination: A Field Manual for Low and Moderate Endemic Countries*.

[2] Federal Democratic Republic of Ethiopia (2012). *National Malaria Guideline*.

[3] Amhara Regional Health Bureau (2011). *Annual Report*.

[4] Protopopoff N., van Herp M., Maes P. (2007). Vector control in a malaria epidemic occurring within a complex emergency situation in Burundi: a case study. *Malaria Journal*.

[5] Regional Office for Africa (2010). *Technical Guidelines for Integrated Disease Surveillance and Response in the African Region*.

[6] Losos J. Z. (1996). Routine and sentinel surveillance methods. *Eastern Mediterranean Health Journal*.

[7] Centers for Disease control and Prevention (2001). Updated guidelines for evaluating public health surveillance systems: recommendations from the guidelines working group. *Morbidity and Mortality Weekly Report*.

[8] Ministry of Health of Ethiopia (2007). *National Malaria Indicator Survey*.

[9] Shama Books (2006). *Ecology of Health and Diseases in Ethiopia*.

[10] Federal Democratic Republic of Ethiopia Population Censes Commission (2008). *Summary and Statistical Report of 2007 Population and Housing Census*.

